# Patient-Reported Outcome Measurement and Reporting for Patients with Soft Tissue Tumors: A Scoping Literature Review

**DOI:** 10.3390/cancers17142280

**Published:** 2025-07-09

**Authors:** Alessandro Mazzocca, Flavia Paternostro, Serena Garofalo, Marianna Silletta, Davide Romandini, Sarah Orlando, Laura Risi Ambrogioni, Pierangelo Gorgone, Giuseppe Tonini, Bruno Vincenzi

**Affiliations:** Medical Oncology, Fondazione Policlinico Universitario Campus Bio-Medico, Via Alvaro del Portillo 200, 00128 Roma, Italy; f.paternostro@policlinicocampus.it (F.P.); serena.garofalo@unicampus.it (S.G.); m.silletta@policlinicocampus.it (M.S.); davide.romandini@unicampus.it (D.R.); sarah.orlando@unicampus.it (S.O.); laura.risiambrogioni@unicampus.it (L.R.A.); pierangelo.gorgone@unicampus.it (P.G.); g.tonini@policlinicocampus.it (G.T.); b.vincenzi@policlinicocampus.it (B.V.)

**Keywords:** quality of life, sarcoma, QoL, GIST, desmoid, TGCT, soft tissue

## Abstract

(1) Background: Quality of life (QoL) assessment is a crucial aspect for patients diagnosed with cancer. Over the years, different tools have been developed to measure QoL, both generic and pathology specific, but the inclusion of quality of life among other indicators of efficacy in randomized controlled trials (RCTs) remains a controversial issue. In this review, we aim to review the frequency and modality of QoL assessment in RCTs, enrolling patients diagnosed with mesenchymal tumors. (2) Methods: An electronic literature search of bone and soft tissue sarcoma and GIST-related RCTs published between January 2000 and December 2023 was performed by two independent reviewers using PubMed. English-language phase II and III clinical trials enrolling at least more than 15 patients were included, regardless of the disease stage. Studies involving patients under the age of 18 years or for which the full text was not available were excluded. For each study, data regarding the journal and year of publication, the study design, the primary objective, and the evaluation of quality of life as an endpoint with any type of patient-reported outcomes used were extracted. (3) Results: Among the 742 publications screened, 171 resulted eligible. QoL assessment was listed among the endpoints in 35 trials and QoL results were reported in 29 primary publications. In these trials, 16 included patients with soft tissue sarcomas, 8 Kaposi sarcomas, 6 GIST, and 3 desmoid tumors. Among all the trials included, 10.4% on an adjuvant/neoadjuvant setting and 24.4% on a metastatic setting included QoL as an endpoint. The proportion of trials, including QoL, was variable over time, as follows: 16.9% of trials in 2000–2014 vs. 23.4% in 2015–2023. In 35 trials, including QoL endpoints, 27 had a superiority design and 25 reported a positive result. In the majority of trials (80%), the tools for QoL assessment were generic and those mostly used were the EORTC QLQ-C30, the EQ-5D questionnaire, and the modified Brief Pain Inventory–Short Form. (4) Conclusions: Quality of life has not been assessed or published in many phase II and III trials, despite an improvement over time. QoL evaluation in RCTs should be considered even more carefully in patients with rare tumors, where the low number of patients who can be enrolled makes it difficult to draw statistically significant conclusions on the effectiveness of treatments.

## Simple Summary

Soft tissue tumors are rare cancers that can cause serious symptoms and affect patients’ quality of life. In recent years, new cancer treatments have been tested in clinical trials, but these studies do not always measure how treatments impact how patients feel and function. This review looked at nearly 25 years of clinical trials on soft tissue tumors to see how often patients’ quality of life was evaluated. The results show that only a small number of studies included this important information, especially in advanced disease or in trials that led to new drug approvals. Most studies used general tools to measure patient experiences, and only a few used questionnaires specific to these rare tumors. These findings highlight a need for more attention to patients’ everyday experiences when testing new treatments. Including quality of life assessments in future research can help doctors make better, more patient-centered treatment decisions.

## 1. Introduction

Soft tissue tumors encompass a group of heterogeneous and rare neoplasms which can be broadly categorized into malignant disease as soft tissue sarcomas (STSs), bone sarcomas, or gastrointestinal stromal tumors, but also locally aggressive benign lesions or with intermediate malignancy, such as tenosynovial giant cell tumors (TGCTs) or desmoid tumors, respectively [1]. These malignancies can occur anywhere in the body, and the therapeutic strategies currently available are based on complex and divergent treatment algorithms [2]. In the majority of cases, for localized disease, the treatment of choice is represented by surgery, often combined with (neo)adjuvant radiotherapy or (neo)adjuvant medical therapy (chemotherapy or targeted therapy), depending on a variety of factors like histology, tumor location, and tumor grade. In some cases, alternative modalities can be considered, such as isolated limb perfusion (ILP) [3], locally advanced or marginally resectable extremity soft tissue sarcomas, or topical treatment, such as in Kaposi sarcomas [4]. On the other side, unresectable, metastatic sarcomas are usually treated with palliative systemic medical therapy and/or radiotherapy [2]. Soft tissue tumors are often diagnosed late due to unspecific symptoms and rare occurrence [5]. Unplanned resections, a result of misdiagnosing the tumor as a more common benign lesion, with a negative influence on the course of treatment, are common [6]. Although malignant soft tissue tumors remain highly lethal diseases, in particular in metastatic patients, survival has improved over time [7]. This progress may be related to the increased knowledge on the clinical and biological characteristics of these tumors and the availability of new drugs and new combination strategies. Notwithstanding the improved life expectancy in these patients, all the aspects that could negatively affect patients’ quality of life should not be forgotten. In fact, soft tissue tumors are usually characterized by a high symptom burden, often leading, also in the case of localized diseases, to physical disabilities and psychological distress, affecting quality of life. Worsening of QoL appears early in sarcoma patients, with a nadir at the time of the surgery, persisting for a long time [8]. However, the health-related quality of life (HRQoL) of sarcoma patients in the different stages of the disease is a rarely investigated topic worldwide [9]. This may be due to the rarity of the disease and because sarcoma patients are treated at different facilities. In this scenario, during the appraising of new treatment options, it is crucial to consider HRQL data in conjunction with efficacy and safety profiles, particularly in those clinical settings characterized by limited life expectancy and a more delicate balance between the benefits and quality of life distress.

Recently, the assessment of quality of life has been increasingly recognized as an essential tool to capture patients’ experience in clinical trials. The patient-reported outcomes (PROs) collected in clinical trials encompass various concepts, including physical functioning, role—physical, bodily pain, general health perceptions, vitality, social functioning, role—emotional, and mental health [10]. PROs, which are outcomes assessed directly by the patient, may produce a different patient’s perspective on the disease and treatment received, complementing the conventional reporting of anti-tumor efficacy data and assisting clinicians in addressing the daily life challenges [11]. Health-related quality of life (QoL) is a specific and multi- dimensional type of PRO related to the physical, psychological, and social impact of the disease and its treatment perceived by patients [12]. In recent years, also scientific societies and regulatory agencies have recognized the value of PROs. The European Medicine Agency (EMA) and the US Food and Drug Administration (FDA) have both provided guidelines for their use, specifically in the setting of oncology clinical trials [12,13]. Although patient-reported outcomes have been explored in several oncology reviews, there is a lack of focused analysis on soft tissue tumors and no comprehensive evaluation of how quality of life is assessed, analyzed, and reported in their clinical trials.

The objective of this scoping review was to map and describe how quality of life endpoints are incorporated and reported in clinical trials on soft tissue tumors published between January 2000 and December 2023. In particular, we analyzed QoL inclusion among study endpoints, the presence of QoL results, and the methodology used for QoL assessment in order to describe methodological patterns and existing gaps that may hinder the integration of patient-centered outcomes into trial design and drug approval processes.

## 2. Material and Methods

**Figure 1:**
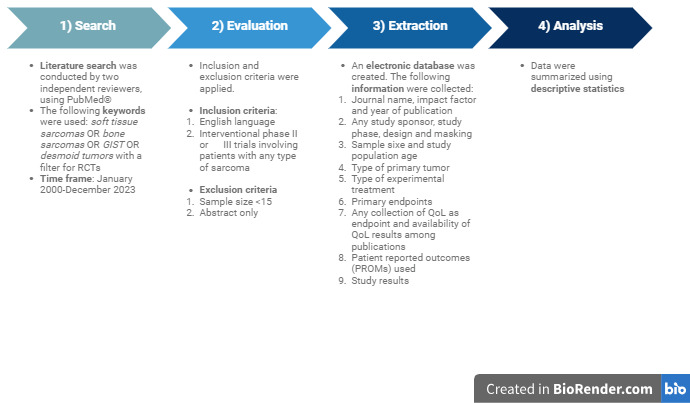
Workflow of the scoping review. GIST: gastrointestinal stromal tumor.

The workflow of our review is summarized in Figure 1. We included, in this analysis, phase II and phase III trials testing different cancer treatment strategies for the treatment of adult patients affected by sarcomas, published in a 24-year time frame between 2000 and 2023. A PubMed search without journal restrictions was conducted in December 2023. The following keywords were used: *soft tissue sarcomas OR bone sarcomas OR gist OR desmoid tumors* with a filter for *Randomized Controlled Trials*.

The predefined inclusion criteria were phase II or III interventional trials testing anticancer treatments in human adult patients with sarcomas, including soft tissue sarcomas, bone sarcomas, gastrointestinal stromal tumors (GISTs), desmoid tumors, and tenosynovial giant cell tumors (TGCTs). The trials testing supportive care drugs or non-pharmacological interventions were not included, unless their outcome was anticancer efficacy. The predefined exclusion criteria were non-interventional trials, as well as trials conducted in malignancy different from sarcomas and trial testing prevention strategies. Both clinical trials that included QoL endpoints and those that did not were analyzed to compare study characteristics and to explore the factors associated with the inclusion of QoL assessments. The trials not reporting QoL data were intentionally included to allow comparison of study characteristics and to identify the patterns associated with the absence of QoL endpoints. This aligns with the objectives of scoping reviews, which aim to map the extent and nature of research activity, including its gaps.

We created an electronic database, including for each trial the following information: journal name, year of publication, impact factor (IF) of the journal at the time of publication, the study sponsor (academic vs. industry sponsored), study phase, number of patients enrolled, type of primary tumor (soft tissue sarcomas, bone sarcomas, bone and soft tissue sarcomas, GIST, desmoid tumors, Kaposi sarcoma, TGCT), disease setting (palliative or adjuvant/neoadjuvant), study design (superiority, non inferiority, non comparative, equivalence), study masking (open label vs. blinded), type of experimental arm (chemotherapy, targeted therapy, immunotherapy, radiotherapy, other), primary endpoint (progression-free survival or disease-free survival, overall survival/OS, tumor response, event-free survival/EFS, other), and study results (positive vs. negative). The information collected about the quality of life (QoL) assessment was as follows: collection of QoL as an endpoint (not included, primary, secondary, or exploratory endpoint), tools adopted for the record of patient-reported outcomes (PROMs) and availability of QoL results (not published, primary, secondary or both primary and secondary publications), and time to secondary QoL publication was calculated from the date of primary definitive publication to the date of secondary QoL definitive publication. Two independent reviewers (AM and FP) screened titles, scrutinized full texts, and extracted data from identified trials. As per the methodological standards of scoping reviews, no formal critical appraisal of the included studies was conducted, since the primary objective was to map the existing evidence rather than assess the risk of bias.

## 3. Results

### 3.1. Study Characteristics

**Figure 2:**
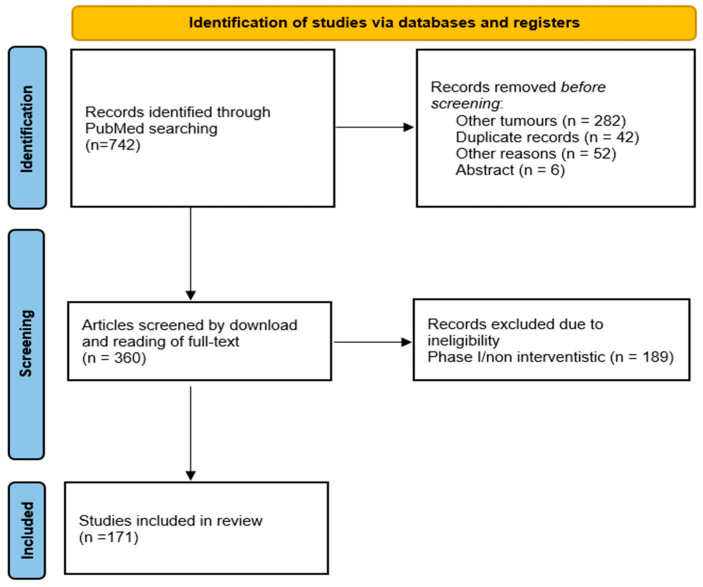
PRISMA flow diagram, depicting the flow of information through different phases of the scoping review.

A PRISMA flowchart of the study selection process is shown in Figure 2. The PubMed search retrieved 742 publications. Overall, 171 publications were fully eligible for this scoping review. The main characteristics of the eligible publications were divided into two time frames (2000–2014 and 2015–2013) and reported in Table 1. Most included studies were published in oncology journals with moderate-to-high impact factors, reflecting the growing editorial attention to QoL endpoints. All the studies were phase II (92, 53.8%) or phase III (79, 46.2%). The trials’ size ranged among less than 50 (30, 17.5%), between 50 and 100 (39, 22.8%), and more than 100 (102, 59.7%) patients. The majority of trials (94, 54.9%) were conducted only in patients with soft tissue sarcomas, GIST (21, 12.3%), and Kaposi sarcoma (21, 12.3%). Most trials (123, 71.9%) were conducted in patients with advanced/metastatic disease in a palliative setting. Chemotherapy (85, 49.7%) and targeted therapy (59, 34.5%) were the most common experimental treatments. The majority of the trials (108, 63.2%) were academic, while the remaining were sponsored by the drug company. In most cases, these trials had a superiority (125, 73.1%) and open-label (136, 79.5%) design.

### 3.2. Inclusion of QoL Among Study Endpoints

**Figure 3:**
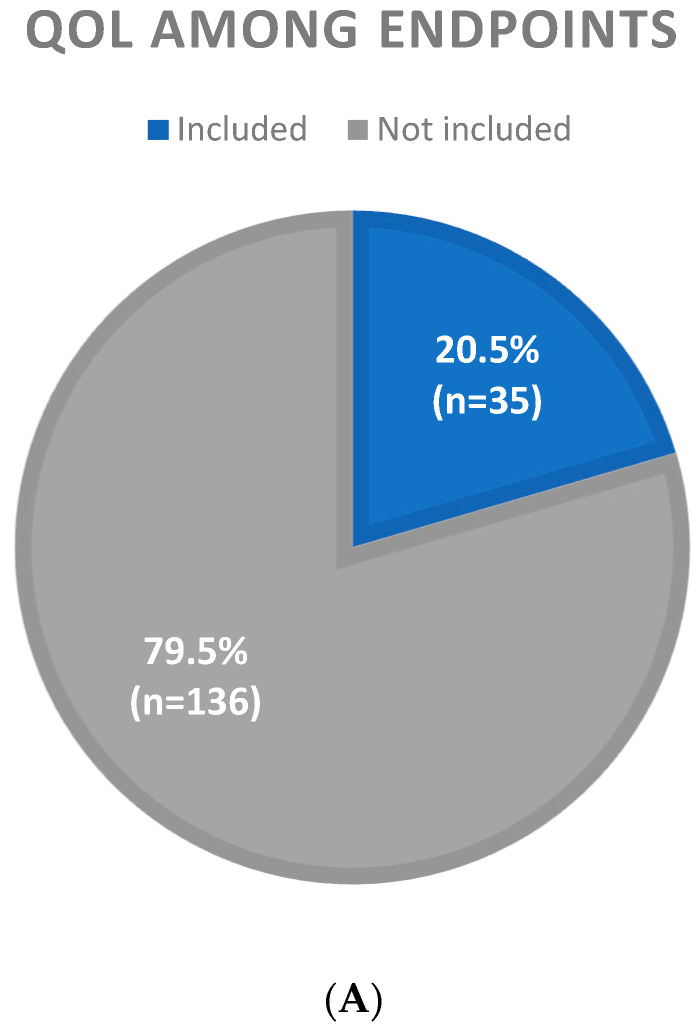

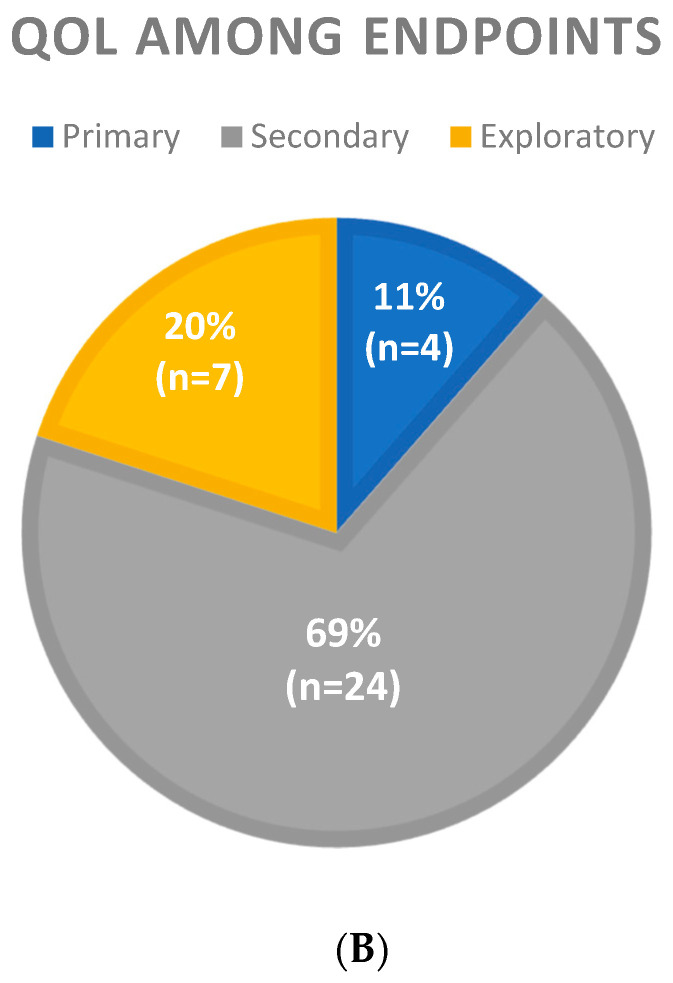
Proportion of phase II and III clinical trials including quality of life (QoL) as a study endpoint.

As shown in Table 2 and Figure 3, QoL was included as an endpoint only in 35 (20.5%) publications. The assessment of quality of life in clinical trials increased over time, with 22/94 (23.4%) of publications including QoL among endpoints between 2015 and 2023 vs. only 13/77 (16.9%) trials in the period of 2000–2014. In particular, QoL was included as an endpoint in more phase III than phase II trials (25.3 vs. 16.3%) and in a higher proportion of industry-sponsored studies (33.4%) compared to academic trials (13%). The inclusion of QoL among endpoints was higher in journals with a high impact factor (38.8%) than intermediate (5.1%) or low (16.9%) and in the subgroup of patients with advanced/metastatic disease (24.4%) compared with those conducted in the localized disease, with only 10.4% of trials exploring quality of life in the neoadjuvant/adjuvant setting. Among different histological subtypes, the QoL assessment was higher in trials on desmoid tumors (75%), Kaposi sarcoma (38.1%), and GIST (28.6%), while it was not listed as an endpoint in an important percentage of trials on soft tissue sarcomas (83%) and bone sarcomas (94.4%). In the majority of trials (24, 69%), QoL assessment was included as a secondary endpoint; in the other 12, it was an exploratory endpoint (7/35, 20%), and only in 4 trials (11%), it was considered a primary endpoint (all phase II studies exploring the role of chemotherapy in Kaposi sarcoma). Most of the trials including QoL assessment reported progression-free survival/disease-free survival (according to the disease setting) or overall survival (OS) as a primary endpoint. As shown in Table 1, studies were classified as negative or positive according to the primary endpoint results. Among the latter, 25 (26.3% included QoL among the endpoints, compared to only 13.4% of the negative ones. An interesting aspect to take into account is that 7/13 (53.9%) of the positive phase III trials, including QoL among endpoints, resulted in Food and Drug Administration approval of the experimental treatment: three of these trials were designed for soft tissue sarcomas, two for GIST, one for desmoid tumor, and one for tenosynovial giant cell tumor, and all were for drugs in the palliative setting (Table 3).

### 3.3. Presence of QoL Results in Primary and Secondary Publications

Out of 35 trials, including QoL among endpoints, QoL results were reported in the majority of primary publications (29/35): in 57% only in primary publications and in 9 cases (26%), both in primary and secondary publications. Most of the trials (19/35) describing the quality of life in primary publications resulted positive for the primary endpoint. In five cases (14%), QoL results were reported not in the primary but only in secondary publications. The median time from the primary to secondary publication was 19 months. In just one case, the quality of life results were published one year before the publication regarding the primary efficacy objective. QoL outcomes were reported in individual trials. Several trials included in this review reported not only the methodology of QoL assessment but also discussed the specific findings in their publications. However, the depth of reporting and interpretation of QoL results varied significantly across studies, and only a few incorporated these data meaningfully in the overall trial conclusions.

Several trials included in this review reported not only the methodology of QoL assessment but also discussed specific findings in their publications. The depth of the reporting and interpretation of QoL results varied significantly across studies. In the *PALETTE* trial, pazopanib was compared to the placebo in pretreated advanced soft tissue sarcoma patients [14]. A secondary analysis focusing on health-related quality of life reported that the QoL scores remained stable over time in both treatment arms [21]. While differences were not large enough to exceed the commonly accepted 10-point threshold for clinical relevance on the EORTC QLQ-C30 scale, the preservation of QoL, despite improved progression-free survival, was considered supportive of pazopanib’s clinical benefit. Similarly, in the *EPAZ* trial, comparing pazopanib to doxorubicin in elderly patients with advanced STS, QoL was assessed using the EORTC QLQ-C30 and remained stable throughout treatment in both arms. Although not statistically superior, the authors emphasized the better tolerability profile of pazopanib and its maintenance of QoL as key considerations in elderly patients with frailty concerns [22]. In the *SAR-3007* phase III trial comparing trabectedin to dacarbazine in patients with advanced leiomyosarcoma or liposarcoma, HRQoL was assessed using the MD Anderson Symptom Inventory (MDASI) [16]. The analysis showed that symptom burden remained stable over time in both treatment arms, indicating that patients treated with trabectedin (or dacarbazine) can maintain good functional performance and low symptom burden for prolonged periods [16]. In the phase III trial evaluating eribulin versus dacarbazine in STS, the HRQoL outcomes were not reported in the main publication [17]. However, a secondary publication reported QoL data, demonstrating that patients receiving dacarbazine had significantly lower global health status and physical functioning scores, along with higher symptom burden compared to those treated with eribulin [23]. These findings suggest that eribulin may be associated with a more favorable QoL profile at progression, complementing its modest survival advantage reported in the main analysis of the trial. In the phase III *GRID* trial, regorafenib was compared to the placebo in patients with metastatic GIST who had failed prior therapy with imatinib and sunitinib [15]. The HRQoL was assessed using the EORTC QLQ-C30. While regorafenib significantly improved progression-free survival, deterioration in global health status, fatigue, and role functioning was observed during treatment. In particular, regorafenib was associated with an increased incidence of hand–foot skin reaction and hypertension, which negatively impacted QoL scores. In contrast, the *INVICTUS* phase III trial evaluated ripretinib versus placebo in patients with advanced GIST who had progressed on at least three prior kinase inhibitors [19]. The HRQoL was assessed using the EORTC QLQ-C30, EQ-5D-5L, and EQ-VAS. The study showed that patients receiving ripretinib experienced a slower rate of decline in physical functioning and global health status compared to the placebo. The authors highlighted that the maintenance of QoL was a key component of the clinical benefit of ripretinib, especially in a heavily pretreated population where disease stabilization and symptom control are primary goals.

Unlike the previously discussed studies focused on malignant soft tissue tumors, where overall survival or progression-free survival represent the main therapeutic goals, tenosynovial giant cell tumor (TGCT) and desmoid tumors represent locally aggressive, non metastasizing mesenchymal neoplasms. In these settings, the burden of disease is more often related to pain, stiffness, and loss of function than to life-threatening complications. As such, patient-reported outcomes and QoL preservation are not only relevant but often constitute the central therapeutic objective. Accordingly, recent pivotal trials in these diseases have incorporated comprehensive QoL assessments as key elements of their clinical evaluations. The ENLIVEN phase III trial evaluated pexidartinib in patients with TGCT. HRQoL assessments using the PROMIS Physical Function scale and a numeric rating scale for stiffness demonstrated that patients treated with pexidartinib experienced significant improvements in physical function and reductions in stiffness compared to the placebo. These benefits were observed as early as week 8 and sustained throughout the study, highlighting the positive impact of pexidartinib on patient QoL [15]. Similarly, the DeFi phase III trial investigated nirogacestat in patients with desmoid tumors. Using a combination of the EORTC QLQ-C30, Brief Pain Inventory–Short Form (BPI-SF), and the desmoid-specific GODDESS questionnaire, the study showed that nirogacestat significantly improved pain, physical functioning, and overall symptom burden compared to the placebo. These improvements were clinically meaningful and underscore the importance of incorporating patient-reported outcomes in evaluating treatments for desmoid tumors.

In summary, while QoL data are increasingly collected, their visibility and interpretive weight in trial publications remain inconsistent. Only a minority of trials discuss QoL findings with the same emphasis as efficacy outcomes, suggesting an ongoing gap between data collection and meaningful integration into clinical decision-making frameworks.

### 3.4. QoL Methodology

Among the included trials that assessed quality of life, the majority (80%) used generic PROMs, while 11.4% employed disease-specific instruments (all in studies on Kaposi sarcoma) and 8.6% incorporated both generic and disease-specific measures (including one study each in Kaposi sarcoma, soft tissue sarcoma, and desmoid tumors). Regarding the specific QoL questionnaires adopted, the EORTC QLQ-C30 was the most frequently used instrument, being employed in 62.9% of studies. Other tools included the EQ-5D (20%), the modified Brief Pain Inventory (mBPI) –Short Form (14.3%), and the EQ-VAS (5.7%). Additionally, 51.4% of studies reported using other assessment tools. These findings indicate a predominance of generic over disease-specific QoL instruments in clinical trials on soft tissue tumors. Details of the QoL methodology in terms of the instruments adopted, are reported in Table 4.

## 4. Discussion

Patients with sarcomas have a significant symptom burden which is slowly progressive and commonly includes pain, fatigue, and dyspnoea [24]. In many cases, particularly for locally aggressive but non-metastatic neoplasms such as desmoid tumors and TGCT, the primary therapeutic goal is not only disease control but also the preservation or improvement of patient-reported quality of life. The previous literature suggests that patients with sarcoma suffer from poorer quality of life (QoL), especially regarding physical and functional wellbeing [21,23]. Recent published reviews acknowledge the paucity of literature on quality of life and psychosocial issues in patients with sarcoma [25]. Given the prevalence of symptoms, potential for treatment toxicity, and poor OS, prospective quality of life data could aid decision making in the sarcoma population with symptom control and quality of life being even more important to consider than life prolongation, particularly in the palliative setting.

Our scoping review demonstrates that a relevant proportion of phase II and III trials evaluating patients with soft tissue tumors did not include QoL among the endpoints, in all disease settings.

When systemic therapy is used as an adjuvant or neoadjuvant option in the early stages of disease, any potential negative impact on quality of life (QoL) may be viewed as temporary and bearable in comparison to the prospect of a permanent cure; however, life expectancy is clearly different in the advanced setting, which accounts for the majority of trials included in the analysis and the majority of patients in clinical practice. The efficacy of systemic therapies remains limited in terms of OS and PFS, while symptoms’ burden can be relevant and the balance between disease control and treatment side effects is far from being obviously positive. In this scenario, it is quite discouraging that in the palliative setting, less than 25% of published trials included QoL among the endpoints. When a trial’s primary endpoint is a surrogate endpoint rather than overall survival, as it is in the majority of trials included in this analysis, QoL assessment becomes even more significant. When looking to single histologies, our analysis revealed that a significant proportion of clinical trials evaluating pharmacological treatments for desmoid tumors and TGCT have incorporated QoL assessments as key endpoints. Unlike the trials conducted for other subtypes, where survival objectives predominate, studies on desmoid tumors and TGCT often prioritize QoL measures given the disease’s impact on functional status, pain phycological well-being, and social functioning. A great proportion (70.3%) of trials with positive results did not include QoL among the endpoints. While the lack of QoL data in a negative clinical trial can be considered almost insignificant, a positive trial represents the first step for a drug to obtain regulatory approval and be included in clinical guidelines and routine clinical practice. In this context, our data are rather disappointing and seem to confirm previous analysis showing that many drugs are approved for marketing without evidence of survival or a quality-of-life benefit [26]. Therefore, we divided trials into profit (when sponsored by a drug company) and no-profit (when sponsored by an academic institution or a cooperative group), and we found in both groups a high proportion of trials not including QoL among endpoints, as follows: 87% among academic trials and 66.6% among industry-sponsored trials. A plausible explanation for the low inclusion of QoL in academic trials can be represented by an increasing awareness among pharma companies of QoL importance in the process of drug approval and reimbursement. On the other hand, academic research faces intrinsic limits related to often inadequate funding, scarce resources, and a shortage of committed personnel, with the result that crucial clinical research elements, such as including quality of life as an objective of clinical trials, must be sacrificed from the outset. Also, we found that trials published in journals with low or intermediate IF included less often QoL among the endpoints rather than high IF journals (QoL was omitted in 83.1%, 94.9%, and 61.2% of trials, respectively). This is reasonable, considering that QoL inclusion could be considered a proxy for trial quality, and high-quality trials are expected to be published, on average, by journals with a higher IF. Although the proportion of trials including QoL among endpoints seems to have a slightly positive trend over time (16.9% in trials published between 2000 and 2014 and 23.4% among those published between 2015 and 2023), the proportion of primary publications reporting QoL results is still low. Therefore, the time that elapsed between primary publications reporting primary endpoints and secondary publications with QoL results is quite long (19 months). Many reasons can underlie these results and explain the delayed reporting of QoL data in secondary publications, after the primary outcome analysis, such as the following: poor compliance, high rate of missing data, and word count limitations imposed by most scientific journals [27]. Obviously, the presence of QoL among endpoints implicates different methodological questions referring to, e.g., the choice of the more suitable type of questionnaire and the best timing of administration [28]. Most of the studies analyzed used generic QoL questionnaires such as EORTC-QLQ-C30, which incorporates different physical, social, functional, and emotional domains [12]. Actually, there is still not a single tool and a comprehensive approach for the assessment of QoL. Unfortunately, with the limited attention dedicated to QoL analysis in the main publications published by the major scientific journals, this future is distant from reality. However, the integration of various methods of analysis could provide a better appreciation of QoL changes. A major goal of QoL assessment in clinical trials is measuring aspects of disease burden that are not fully represented by parameters like disease stage, comorbidities, or performance status alone. Physical functioning and performance status could be viewed as assessments of the same complex situation, but from different angles, and some evidences seems to suggest that the examining physicians might overlook some subjectively reported toxicities [29]. Moreover, another aspect to take into account and further explore is also the potential independent prognostic value of HRQoL domains in sarcoma patients [30]. Trials incorporating QoL endpoints provide valuable information that can guide shared decision-making, allowing clinicians to balance treatment efficacy with symptom burden and functional impairment. Conversely, the absence of QoL data limits the ability to fully assess the patient-centered benefits of new therapies, especially in diseases where survival endpoints are not the sole priority. Future clinical trials should ensure the early integration of QoL endpoints as core elements of trial design, especially in rare tumor settings where symptom control often outweighs survival gain. This includes selecting validated, disease-specific PROMs when available and clearly defining the timing and endpoints for QoL assessments, ensuring an adequate sample size for PRO analyses and a timely publication of results. Collaboration with QoL methodologists and patient advocacy groups is also recommended to improve relevance and completeness of reporting.

### Strenghts and Limitations

This scoping review represents, to our knowledge, the first structured mapping of how quality of life (QoL) endpoints are incorporated and reported in clinical trials focusing on soft tissue tumors, including gastrointestinal stromal tumors, desmoid tumors, and TGCT. By systematically analyzing trials over a defined time frame, we provide a comprehensive overview of the current practices in QoL assessment in this rare and heterogeneous group of diseases. The inclusion of studies across different tumor subtypes and therapeutic strategies adds breadth to the findings, and the use of predefined criteria ensures methodological transparency. Anyway, several limitations must be acknowledged. First, the literature search was restricted to PubMed, which may have resulted in the omission of relevant studies indexed in other databases. Second, as per the methodological standards of scoping reviews, no formal critical appraisal of study quality was performed. Third, the heterogeneity of trial designs, patient populations, and QoL instruments prevented quantitative synthesis or meta-analysis. Lastly, the heterogeneity of tumor types included in this review represents an inherent limitation. In fact, the variability in surgical approaches, systemic therapies, trial phases, and instruments used for QoL assessment introduces a significant degree of variability that limits a direct comparability of outcomes. Moreover, these neoplasms differ substantially in biological behavior, therapeutic strategies, and prognosis. However, the inclusion of different histotypes, particularly locally aggressive mesenchymal tumors such as desmoid tumors and TGCT, was considered appropriate given that their clinical management overlaps with soft tissue sarcomas and the central role of quality-of-life outcomes in treatment decision-making for these diseases. However, this diversity reflects the real-world complexity of managing soft tissue tumors and highlights the need for methodological standardization.

## 5. Conclusions

Health-related quality of life should be integrated into tailoring treatment selection in soft tissue tumor patients as patient-reported outcomes are a key element of shared decision- making and patient-centered care. Toxicity assessment of specific treatments in clinical trials should not be a surrogate measure for the quality of life of patients, and careful assessment of global QoL scores, as well as individual symptom scores, may provide key insights into the impact of the disease on quality of life and functioning. QoL evaluation in clinical trials should be considered even more carefully in patients with rare tumors, where the low number of patients who can be enrolled makes it difficult to draw statistically significant conclusions on the effectiveness of treatments, and it should also be extended to real-life studies in order to identify the real impact of therapy choices on patients living with sarcomas. Finally, incorporating QoL endpoints as primary or co-primary outcomes should be seriously considered in future trials, particularly for non-metastasizing but disabling conditions like desmoid tumors and TGCT, where therapeutic success is defined more by symptom relief than by tumor shrinkage.

## Figures and Tables

**Table 1:** Characteristics of the 171 primary publications included in the analysis.

	2000–2014 *n* = 77 (45.0%)	2015–2023 *n* = 94 (55.0%)
**Study sponsor**		
Academic	52 (67.5%)	56 (59.6%)
Industry sponsored	25 (32.5%)	38 (40.4%)
**Study phase**		
II	44 (57.1%)	48 (51.1%)
III	33 (42.9%)	46 (48.9%)
**Study design**		
Superiority	57 (74.0%)	68 (72.3%)
Non comparative	12 (15.6%)	14 (14.9%)
Non inferiority	5 (6.5%)	6 (6.4%)
Equivalence	3 (3.9%)	6 (6.4%)
**Masking**		
Open label	64 (83.1%)	72 (76.6%)
Blinded	13 (16.9%)	22 (23.4%)
**Tumor type**		
Soft tissue sarcomas	38 (49.3%)	56 (59.6%)
GIST	11 (14.3%)	10 (10.5%)
Bone sarcomas	6 (7.8%)	12 (12.9%)
Soft tissue + bone sarcomas	4 (5.2%)	8 (8.5%)
Desmoid tumor	0 (0.0%)	4 (4.2%)
Kaposi sarcoma	18 (23.4%)	3 (3.2%)
Tenosynovial giant cell tumor	0 (0.0%)	1 (1.1%)
**Disease setting**		
Palliative	51 (66.2%)	72 (76.6%)
Adjuvant/Neoadjuvant	26 (33.8%)	22 (23.4%)
**Experimental treatment**		
Chemotherapy	47 (64.0%)	38 (40.4%)
Targeted therapy	15 (19.5%)	44 (46.8%)
Immunotherapy	1 (1.3%)	7 (7.5%)
Radiotherapy	5 (6.5%)	3 (3.2%)
Other	9 (11.7%)	2 (2.1%)
**Primary endpoint**		
PFS/DFS	22 (28.6%)	47 (50.0%)
Tumor response	25 (32.5%)	16 (17.0%)
OS	14 (18.1%)	7 (7.5%)
EFS	5 (6.5%)	13 (13.8%)
Other	11 (14.3%)	11 (11.7%)
**Study results (primary endpoint)**		
Positive	43 (55.8%)	52 (55.3%)
Negative	34 (44.2%)	42 (44.7%)

Abbreviations: GIST, gastro intestinal stromal tumor; PFS, progression-free survival; DFS, disease-free survival; OS, overall survival; EFS, event-free survival.

**Table 2:** Inclusion of health-related quality of life (QoL) among studies’ endpoints.

	QoL Not Included n = 136 (79.5%)	QoL Included n = 35 (20.5%)
**Year of primary manuscript**		
2000–2014	64 (83.1%)	13 (16.9%)
2015–2023	72 (76.6%)	22 (23.4%)
**Study sponsor**		
Academic	94 (87.0%)	14 (13.0%)
Industry sponsored	42 (66.6%)	21 (33.4%)
**Study phase**		
II	77 (83.7%)	15 (16.3%)
III	59 (74.7%)	20 (25.3%)
**Study design**		
Superiority	98 (78.4%)	27 (21.6%)
Non comparative	20 (87.0%)	3 (13.0%)
Non inferiority	9 (81.8%)	2 (18.2%)
Equivalence	6 (66.6%)	3 (33.4%)
**Masking**		
Open label	112 (82.3%)	24 (17.7%)
Blinded	24 (68.6%)	11 (31.4%)
**Tumor type**		
Soft tissue sarcomas	78 (83.0%)	16 (17.0%)
Bone sarcomas	17 (94.4%)	1 (5.6%)
GIST	15 (71.4%)	6 (28.6%)
Kaposi sarcoma	13 (61.9%)	8 (38.1%)
Soft tissue + bone sarcomas	12 (100%)	0 (0.0%)
Desmoid tumor	1 (25.0%)	3 (75.0%)
Tenosynovial giant cell tumor	0 (0.0%)	1 (100%)
**Disease setting**		
Palliative	93 (75.6%)	30 (24.4%)
Adjuvant/Neoadjuvant	43 (89.6%)	5 (10.4%)
**Experimental treatment**		
Chemotherapy	71 (83.5%)	14 (16.5%)
Targeted therapy	43 (72.9%)	16 (27.1%)
Immunotherapy	7 (87.5%)	1 (12.5%)
Radiotherapy	5 (62.5%)	3 (37.5%)
Other	10 (90.9%)	1 (9.1%)
**Primary endpoint**		
PFS/DFS	51 (73.9%)	18 (26.1%)
Tumor response	36 (87.8%)	5 (12.2%)
OS	16 (76.2%)	5 (23.9%)
EFS	17 (94.4%)	1 (5.6%)
Other	16 (72.7%)	6 (27.3%)
**Study results (primary endpoint)**		
Positive	70 (73.7%)	25 (26.3%)
Negative	65 (86.6%)	10 (13.4%)

**Table 3:** Phase III clinical trials with QoL among endpoints and positive results leading to drug regulatory approval.

Author/Year	Disease	Experimental Treatment	Control Treatment	Primary Endpoint	QoL Endpoint	QoL Methodology	QoL Results
van der Graaf WTA et al., 2012 [14]	STS	Pazopanib	Placebo	PFS	Secondary	EORTC QLQ- C30, EQ-5D, Global Heath status/quality-of-life score	Primary and secondary publication
Demetri GD et al., 2013 [15]	GIST	Regorafenib	Placebo	PFS	Exploratory	EORTC QLQ-C30, EQ-5D	Secondary publication
Demetri GD et al., 2016 [16]	STS	Trabectedin	Dacarbazin	OS	Secondary	M.D. Anderson Symptom Inventory (MDASI)	Secondary publication
Shoffski P et al., 2016 [17]	STS	Eribulin	Dacarbazin	OS	Exploratory	EORTC QLQ-C30, EQ-5D	Secondary publication
Tap WT et al., 2019 [18]	TGCT	Pexidartinib	Placebo	Tumor response	Secondary	PROMIS, stiffness, Brief Pain Inventory, Pain-30	Primary and secondary publication
Blay JY et al., 2020 [19]	GIST	Ripretinib	Placebo	OS	Secondary	EORTC QLQ-C30, EQ-5D-5L, EQ-VAS	Primary and secondary publication
Gounder M. et al., 2023 [20]	Desmoid Tumor	Nirogacestat	Placebo	PFS	Secondary	EORTC QLQ-C30, Brief Pain Inventory, GODDESS, DTSS, DTIS	Primary and secondary publication

STS: soft tissue sarcoma; GIST: gastrointestinal stromal tumor; TGCT: tenosynovial giant cell tumor; EORTC QLQ-C30: European Organization for the Research and Treatment of Cancer quality of life questionnaire; EQ-5D: EuroQol Group 5-dimension; Pain-30: brief pain inventory-30 definition; PROMIS: Patient-Reported Outcomes Measurement Information System-Physical Function scale; QoL: quality of life; EQ-VAS: EuroQol Visual Analogue Scale; GODDESS: Gounder–Desmoid.

**Table 4:** Details of methodology of quality-of-life assessment.

	N (%)
**Type of PROMs**	
Generic	28 (80%)
Disease specific (*all in Kaposi Sarcoma*)	4 (11.4%)
Both generic and disease specific (*1 in KS, 1 in STS, 1 in desmoid tumor*)	3 (8.6%)
**QoL questionnaire (not mutually exclusive)**	
EORTC QLQ-C30	22 (62.9%)
EQ-5D	7 (20%)
mBPI short form	5 (14.3%)
EQ-VAS	2 (5.7%)
Other tools	18 (51.4%)

KS: Kaposi sarcoma; STS: soft tissue sarcoma; EORTC QLQ-C30: European Organization for the Research and Treatment of Cancer quality of life questionnaire; EQ-5D: EuroQol Group 5-dimension; mBPI: modified brief pain inventory; EQ-VAS: EuroQol Visual Analogue Scale; QoL: quality of life.
